# Membrane voltage multistability in coupled glial cells

**DOI:** 10.64898/2026.05.03.722503

**Published:** 2026-05-08

**Authors:** Predrag Janjic, Dimitar Solev, Min Zhou, Ljupco Kocarev

**Affiliations:** 1 Research Centre for Computer Science and Information Technologies, Macedonian Academy of Sciences and Arts, Skopje, North Macedonia,; 2 Department of Neuroscience, Ohio State, University Wexner Medical Center, Columbus, Ohio, USA

## Abstract

Growing interest to describe the electrical behavior of glial cells, mainly astrocytes, in intact brain tissue poses more and more challenges to commonly accepted belief they only respond in a linear manner in uptake of the excess of extracellular potassium and maintenance of their network equipotentiality. Their highly conductive mutual interconnections via gap junction (GJ) connections introduce yet another class of nonlinear elements. As more studies report nonlinearities in membrane voltage $V_{m}$ dependence of both, the membrane and junctional conductances, the need to formulate minimal dynamical models of their transient behavior is getting more acute. Since ODE models of coupled cells, even in simplest 1-d arrays, require simplified descriptions and small set of parameters, rare quantitative studies on glia makes the task even more difficult. This study attempts to qualify a *self-coupled cell*, or a glial cell coupled to fixed voltage as useful system for detecting the nature of instabilities and transitions coming from coupling. In a novel biophysical model of coupled astrocyte, we introduce nonlinear kinetics of deactivation for large junctional voltages for the first time. We found that N-shaped nonlinearities and corresponding fold structure in the vector field of isolated cell serves as a baseline on top of which coupling nonlinearities enrich the bifurcation picture. Numerical simulations of 1-d array of coupled astrocytes show that coupling increases the propensity of astrocytic $V_{m}$ to bistability and front propagation. We believe that presented illustrations of possible effects of coupling nonlinearities will motivate neurobiologists to further explore their impact in disease.

Neuronal cells of the brain and spinal cord cannot exist and function without supportive and neuromodulatory functions of the diverse population of glial cells. This applies to virtually all physiological processes on cell level - from cell development, metabolic support, membrane signaling, slow molecular signal transduction, ion homeostasis, neurovascular coupling, myelination, to mention only a few, manifest neuro-glial interaction. Even though all glial cell types are interconnected, the most abundant ones, the astrocytes are massively interconnected by gap junctions to form ordered networks. Electrically, astrocytic networks display membrane voltage equipotentiality, which is considered system-wide resting state for given neuro-glial circuit or unit. With molecular and cellular substrates of glial connectivity being slowly elucidated, network science and dynamical modeling are slowly “invading” that area with many important issues left open. In this study using classical dynamical systems approaches we give indications how nonlinear intercellular coupling between astrocytes affects physiological resting state and its instabilities compared to isolated, uncoupled cell. We strongly believe the suggested minimal model could fill the gap in ODE modeling of neuro-glial circuits, within broadest scope of hypothesis-driven research in cell-level neuroscience.

## 1. Introduction

**Figure 1. –:**
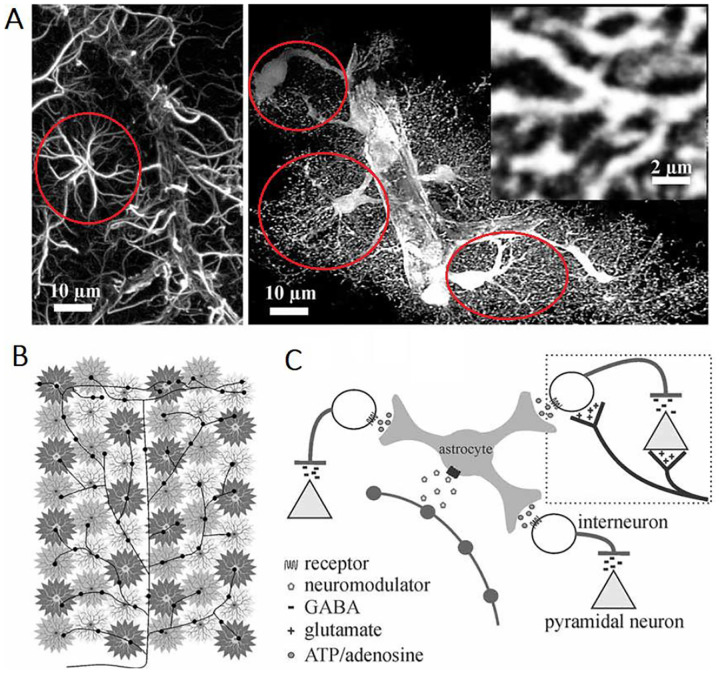
Network features of astrocytes – **(A)** (left) Star-like morphology of astrocytes (circled red) from the rat cortex, where using the traditional immunolabeling outlines only major branches. (right) In contrast, when using enhanced fluorescence, the astrocytes reveal their highly complex and delicate morphology. Original source images adapted from Simard and Nedergaard (2004) with permission. **(B)** Network of interconnecting astrocytes (gray stars) interacts in multiple ways with elements of directly apposing neuronal circuits. **(C)** Typical model of astrocytic role in neurotransmission and neuromodulation acting in excitation-inhibition control of local canonical circuits, shown simplified in (B). Figure adapted from (Pacholko et al.,2020)^6^, with permission.

The glial cells, especially astrocytes, represent a diverse class of neural cells that play many roles in neural function, directly interworking with the neurons and blood vessels^1^. The crucial systemic feature of astrocytes is their massive interconnection through the extensively branching processes into highly conductive networks, treated like a form of cellular continuum, called *syncytium*^2,3^. The elongated, extensively branching fine peripheral glial processes^4^ allows close spatial integrated into the brain networks^5^. Figure 1A shows some visualizations of these very specific structural properties of glia which in turn undoubtedly define their function(s) in great part. Besides the increasing focus on glial cells in all domains of neuroscience, dynamical modeling has not yet endorsed the glial networks.

Modeling glial $V_{m}$ dynamics, it is important to state that the electrical properties of the glial membrane are very similar to those of the neuronal membrane^7^, mainly lacking transient voltage-gated sodium and calcium channels which deprives them from spiking. In addition to being the main control variable, membrane voltage $V_{m}$ acts in the same time as target variable of various neuromodulatory circuits (Fig. 1C). Since in all neural cells near the resting membrane voltage (RMV), where only potassium and chloride channels are permeant, the resting voltage $V_{r}$ in glia is close to that one in neurons, but 10 to 15mV more negative, lying between -80mV and -70mV, near $K^{+}$ and ${Cl}^{-}$ Nernstian reverse potentials^7^. That is where several of the transporters have their nominal operational range, engaging glia in the “housekeeping” of transient ionic imbalances, resulting from sustained neuronal activity. The $V_{r}$ stability is dominantly controlled by the ATP-driven sodium-potassium ion pump (NKA)^8,9^ through maintenance of the Nernstian reverse potential. Glial NKA displays electrogenic properties specific for glia - different from the NKA pump found in most of the neurons^9,10^, and significant for the differential role of glia in ion homeostasis.

Since electrical processes within neural cells are under dynamical control of the membrane voltage, $V_{m}$ stability has a profound effect not only on the electrogenic phenomena through the membrane, but on most functions of the cell. In a recent modeling study^11^ we showed that when a specific mix of different subtypes of dominant glial $K^{+}$ currents – predominantly the Kir (potassium inward rectifier) and K2P (potassium two-pore domain) currents, or their deregulation introduce N-shaped nonlinearity within I-V curve, an isolated glial cell could be prone to *bistability switching*, when perturbed with seizure-like transient changes of the immediate local field potential (LFP).

Today, computational studies of neuro-glial network interaction is a subject of growing focus, exploring a very wide scope of structural and functional details, see (Manninen et al, 2023)^12^ for review. To which extent the *synchrony within glial* networks parallels the synchrony of neuronal networks is one of the central questions quantitative neuroscience modeling is trying to address. Even though compartmental models of glia exist, they have mainly focused on the glial role in the regulation of potassium homeostasis, using trivial models of coupling, even though the GJs have been extensively studied in other cell populations.

Since most glia, especially the astrocytes are heavily interconnected among them by nonlinear GJ connections it makes an isolated astrocyte only a *building block* of a more realistic neural assembly. To address this gap, in this study, we develop a minimal low-dimensional model of a coupled glial cell, incorporating both voltage dependence of GJ conductance, as well as GJ activation kinetics of all junctions. Under common simplifying assumptions we performed limited parametric analysis on a “truncated” system – *self-coupled* glial cell, representing an inner cell within 1-d array of coupled glia, as a simplest cell array model. Several bifurcation scenarios presented in more detail illustrate various forms of $V_{m}$ multistability caused by coupling. Numerical simulations of a small 1-d array illustrate that nonlinear GJ coupling impact the $V_{m}$ dynamics governing the propagation or failure of instabilities along the array.

## 2. Glial coupling

All glial cells form interconnected networks.^2,13^ In most brain regions astrocytes are in particular massively interconnected^2^, Fig. 1A, on average with 7–10 immediate neighbors^14^ by direct electrical junctions formed by *gap junction channels* (GJCs). Electrically, in physiological conditions these networks are *isopotential*^14^ – trying to maintain near the same $V_{m}$ with a capability to swiftly restore the disturbances of such state^15^. Physically, such massively interconnected continuum is formed by the junctions located close to the tips of the astrocyte branches.^4^ The junctions are formed by clusters of hundreds up to two thousands GJCs, in a single membrane cluster^16^ or *plaque, see* Supplementary Material S1 for more details on the build-up of a single gap junction. The physical GJ sizes and channel densities within a single glial gap junction^4^ suggest a complexity that cannot be conceptualized with a single channel pore, Fig. S1C. For more structure-function details on GJ connections see (Sosinsky G., 2000)^17^.

### 2.1 Passive electrical properties of gap junction connections

Two-electrode whole-cell recording has been a standard protocol for measuring steady-state conductance of GJs between two cells. However, such a setup is very limiting for assessing passive electrical properties of *a single whole junction*, and in turn quantitating the steady-state or properties. The measuring circuit records only from two terminals, summing up contributions of all junctions in readouts of single current, offering just the total conductance as coming from a single, “*mega*” junction. As a result, where both *instantaneous* and *steady-state* I-V responses are reported, differentiating the kinetical effects of larger compared to the smaller junctions is out of experimental reach.

Distinct molecular buildup of the GJ introduces *cytoplasmic access resistance*, $R_{\text{cyt}}$, which physically does not simply represent a fraction of the whole cell input resistance $R_{m}$, but resistance by the passive resistive properties of the whole GJ as a “connector device”^18^. For the equivalent electrical circuit of a whole GJ, see Supplementary Material S2. Considering the passive electrical properties, the junction represents a highly conductive (low-resistance) macroscopic connection or a *bridge*, variable in size, introducing specific electrical properties as a distinctive membrane domain, Fig. S1C, Supplementary Material S1. Further to the resistance/conductivity variations, the junction introduces local capacitance variation, and possibly a new time constant describing the *polarization of hundreds to thousands of channels*. In circuit terminology, abandoning the implicit assumption of an electrically small cell, the GJ-specific electrical polarization would mean introducing a GJ-specific *capacitive transient* polarizing the plaque. This requires evaluating the range of that time constant, or variations of membrane capacitance $C_{m}$. In other words, a time constant $\tau_{gj}$ specific to the plaque polarization should be considered describing the whole junction (de)activation kinetics with $V_{j}$. Dynamically, this indicates likely difference between momentary and steady-state response in large junctions over different time scales.

**Figure 2. -:**
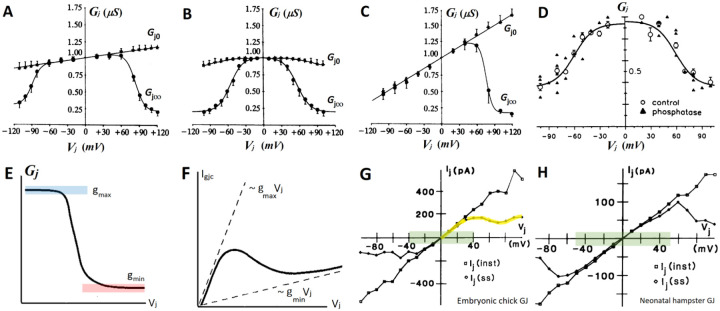
Instantaneous vs. steady-state voltage dependence of GJ conductance – **(A)-(C)** Voltage dependence of homotypic Cx26/Cx26, Cx32/Cx32, and heterotypic Cx26/Cx32 gap junctions respectively, showing marked difference between the instantaneous $G_{j0}$ (filled asterisks) and steady-state conductance $G_{j\infty }$, (filled circles), figures modified from (Barrio, 1991)^30^ with permission. **(D)** Normalized $G_{j}-V_{j}$ dependence of GJs formed by Cx43 gap junctions, showing high normalized residual conductance $g_{\text{min}}$, a feature of homotypic Cx43 junctions dominating in glia, figure from (Moreno 1992)^34^ with permission. All steady-state dependences in (A)-(D) are fitted with the Boltzmann equation of first-order activation kinetics, with residual conductance $g_{\text{min}}$. **(E)** A sketch of single-sided Boltzmann relation with a residual term approximating the profiles in (B) and (D), and **(F)** the corresponding $I_{gjc}-V_{j}$ current profile with the typical N-shaped nonlinearity, sketches modified from (Baigent, 2001)^35^, with permission. **(G), (H)** Instantaneous (squares) and steady-state (rhombs) current in gap junctions from chick embryo and neonatal hamster closely reflecting the qualitative shape of the voltage dependence of conductances in (A)-(D), modified from (Veenstra, 1992)^21^ permission requested from T&F via form. The $V_{j}$ range in which we observe plateau in (A)-(D) gives the $V_{j}$ range (shaded green) where measured $I_{gjc}$ currents closely follow each other. The positive N-shaped branch of $I_{gjc}$ current in (G), shaded yellow, qualitatively matches the sketch of the steady-state approximation in (F).

For the single GJ channel conductance, rather wide range of estimates have been reported, between 50÷140pS^19–21^ for glial-specific GJC subtypes^22^ Cx43 and Cx30. Whole-cell modeling studies based on measurements on native GJCs in glia^14^ have suggested ${\overset{‾}{g}}_{gjc}=120\;pS$ as estimate of fully conducting single GJC, estimated using multiple gates kinetical model^20,23^. Two gating states correspond to the two distinct permeation states - *fully conductive* and *residual* (partly conductive) state,^24,25 25^ the latter estimated at 20% to 40% of the maximal conductance, Fig. 2A to 2D. Once the molecular details of the Connexin channels and single GJC pore were revealed^26^ - the large, contiguous open pore, over 15nm long and 1.5-2nm wide^17,27^ with low ion selectivity, could justify the very large conductances and low selectivity of GJCs.

In the formulation of the coupling model, we will adopt the first-order GJC kinetics as suggested by A. Harris and coworkers in the legacy studies on early embryonic amphibian cells (*blastomeres*)^28,*29* 29^.

For average conductance of whole GJ we will use an estimate from (Kiyoshi et al, 2018, see Suppl. Information)^15^ of 2,000 GJ channels between two directly connected cells, setting an upper limit of 240 nano siemens per GJ.

### 2.2 Voltage dependence of gap-junction conductance

Observing that fully assembled GJC channel consists of two separate channels on each membrane, we recognize different voltage dependencies of the single GJC and GJ conductance: a $V_{m}$-dependence, representing conductance dependence on the membrane voltage $V_{m}$ of each of the channels, as well as $V_{j}$-dependence on the transjunctional voltage $V_{j}$, or $V_{m}$ difference between two connected cells. Even though the former has been demonstrated experimentally^30^, its effects could not be described physically, because the permeability of such a *half-GJC* cannot be measured. Fortunately, only in some of the Connexins we see very slight impact of different steady-state $V_{m}$ values, with a minor impact on $V_{j}$-gating of functional GJC channel^25,30,31^. In what follows by voltage dependence, we will only refer to dependence on the transjunctional voltage $V_{j}$, which for cell-A and cell-B we define as $V_{j}=V_{m,A}-V_{m,B}$, by convention taken positive as $\left(V_{m,\text{OTEHR}}-V_{m,\text{OWN}}\right)$. In the rest of our model development, we will also pass from apparent resistance to conductances, as conventional in conductance-based description of the neural circuits.

Two well-established experimental properties define our whole-cell model of glial coupling, **(i)** a *time-dependent kinetics* - distinctive difference between the instantaneous and steady-state dependence of the junctional conductance $g_{gj}$ for large $V_{j}$, Fig. 2A–2C, and **(ii)** voltage-dependent GJ deactivation. The kinetics of whole GJ is governed by deactivation time constant $\tau_{gj}$, which we already defined as time constant of polarization of the GJ proportional to its size. This does not exclude GJ activation, but two-electrode recordings have shown that it is very fast, typically less than 10 milliseconds^3,15,32^. Since two-electrode recordings sum up the currents from all GJ connections between two cells, they also average the *size effect* – dependence of the junction kinetics on the physical size and number of channels in each junction^18,33^, justifying thus the use of a single $\tau_{gj}$. In the absence of measurements constrained on *single* junctions in native cells, we model the resistive properties of the junctions as arising from a sum of junctions with average number of channels and plaque size^18^.

## 3. Mathematical model of glial coupling

The Vogel-Weingart gating models^20,36^ and the legacy studies by Harris, Spray, and Bennett^28,29,37^, as well as those who have adopted the later model like Verselis and Bennett with coworkers^38^, have not introduced *time-dependent kinetical parameters* on the level of a whole junction. Instead, the mentioned studies all adopted the Boltzmann equation for open/close kinetics, leaving the GJ size effects in $V_{j}$ dependence and its timescale out of the initial model.

To narrow towards GJ-level model, let’s state the following assumptions on the model system:

- Instantaneous conductance in glial *homotypic* junctions is near constant, almost voltage-independent over a wide $V_{j}$ range, Fig. 2B and 2D. For small $V_{j}$ near zero it reaches maximal value $g_{\text{max}}$, dominated by the fully open conformation in most of GJCs. This implies that nonlinearity in steady-state $g\left(V_{j}\right)$ dependence describes ***deactivation of GJ*** as $V_{j}$ increases in absolute value beyond the knee of plateau curves, Fig. 2A to 2E). Since there is no stable closed confirmation, with $V_{j}$ close to zero, we consider both half-GJCs open and the GJC fully conductive.
- In whole-cell models with GJ coupling, the junction inactivation is described by a first-order differential kinetic variable $n_{gj}$, with steady state deactivation $n_{\infty }\left(V_{j}\right)$ based on $g\left(V_{j}\right)$.
- The time constant observed in studies of both, single channels and whole junctions, represents a time constant of deactivation, $\tau_{gj}$ in first-order open-close transitions (not *vice versa*). It reflects junction-specific polarization time, needed for membrane polarization to reach the innermost GJCs channels propagating from the rim of the plaque toward the center^33^, when conductance drops to $g_{\text{min}}$, Fig. 2E.
- The residual conductance of any single GJC, $\gamma_{\text{res}}$ is independent of the voltage and reflects the properties of the specific connexin subtype(s). It will be used in estimates of *residual GJ conductance* $g_{\text{min}}$, when fitting Eq. (1) below, numerically.

Under these assumptions, we adopt Boltzmann equation for two-state, voltage-dependent steady-state deactivation probability $n_{\infty }\left(V_{j}\right)$, observed in all GJ channel subtypes, to write:^37^

(1) $$n_{\infty }\left(V_{j}\right)=g_{\text{min}}/\left(g_{\text{max}}-g_{\text{min}}\right)+1/\left(1+e^{-\left(V_{j}-V_{j12}\right)/v_{s}}\right),\;=n_{\text{res}}+1/\left(1+e^{\left(V_{j}-V_{j12}\right)/v_{s}}\right),\;\Delta g_{j}\equiv g_{\text{max}}-g_{\text{min}},\;n_{\text{res}}\equiv \frac{g_{\text{min}}}{\Delta g_{j}},$$

where $g_{\text{max}}$ and $g_{\text{min}}$ represent the maximal and residual conductance, $V_{j12}$ the half-activation voltage, while $v_{k}\equiv RT/F$ is the thermal slope factor equal to 25.7mV at room temperature of 25°C.

Estimating maximal $\tau_{gj}$ within 0.4 to 0.6 seconds range^16,18,28^ suggests timescale separation of at least two orders of magnitude compared to membrane time constant $\tau_{m}$, typically up to 10 milliseconds representing average time of cell polarization assuming momentary activation of whole-cell conductances of $K^{+}$ channels. Predominantly expressed Kir channels are usually not modeled kinetically due to almost instantaneous activation within one millisecond. According to assumption (iii), this implies introducing a separate ODE variable for $n_{gj}$ to account for much longer $\tau_{gj}$

Extending the original minimal model of a single astrocyte^11^ with a generalized coupling current $I_{gj}$, it becomes:

(2) $$C_{m}dV_{m}/dt=I_{\text{ext}}-\left(I_{Kir}+I_{K2P}+I_{\text{leak}}\right)+I_{gj}\left(V_{m},V_{j}\right)\;(nA),\;dn/dt=\left(n_{K2P}\left(V_{m}\right)-n\right)/\tau_{K2P},\;dn_{gj}/dt=\left(n_{\infty }\left(V_{j}\right)-n_{gj}\right)/\tau_{gj}.$$

Since with $\tau_{K2P}\approx 3\;ms$ activation of K2P current is almost instantaneous compared to junctional $\tau_{gj}$, we can drop the second ODE, at least in qualitative analysis of the stability of $V_{m}$ steady states, while keeping only GJ deactivation kinetics $n_{gj}$.

Without consensual range estimates of $\tau_{gj}$, for simplicity we kept it constant at $\tau_{gj}=100\;ms$. Large errors in this estimate could impact only jump transition time in simulations, but not the ODE stability analysis.

### 3.1 One-dimensional array of coupled astrocytes

The simplest non-biological constructs traditionally used in models of coupled cells are the 1-d arrays of N cells coupled by GJs. After removing the fast K2P current kinetics (the 2^nd^ ODE in (2)), for an inner cell coupled to two immediate neighbors in the 1-d array, we could write:

(3) $$C_{m}dV_{m}/dt=f\left(V_{m},I_{\text{ext}}\right)+l_{\text{scal}}{\overset{‾}{g}}_{gj}n_{gj}\left(V_{m}^{l}-V_{m}\right)+r_{\text{scal}}{\overset{‾}{g}}_{gj}n_{gj}\left(V_{m}^{r}-V_{m}\right)\;(nA),\;dn_{gj}/dt=\left(n_{\infty }\left(V_{j}\right)-n_{gj}\right)/\tau_{gj},\;f\left(V_{m},I_{\text{ext}}\right)=I_{\text{ext}}-\left(I_{Kir}+I_{K2P}+I_{\text{leak}}\right)$$

where ${\overset{‾}{g}}_{gj}$ represents the total slope conductance of all gap junctions in the cell, divided in $l_{\text{scal}}$ and $r_{\text{scal}}$ fractions between the two neighbors, and $V_{m}^{l}$ and $V_{m}^{r}$ the membrane voltages of the left and right neighbor, Equation (3) representing the i-th cell, 2≤i≤N-1 is not a proper, complete model since we are missing the ODEs for the neighbors. The $V_{m}$-dependence of f is hidden behind $I_{Kir},I_{K2P}$ and $I_{\text{leak}}$ which are all voltage-dependent. We will later discuss the rationale behind the segregation of GJ connections using $l_{\text{scal}}$ and $r_{\text{scal}}$. For detailed biophysical description of $I_{\text{Kir}}$ and $I_{K2P}$ see the isolated cell model^11^.In the classical differential-difference reaction-diffusion (RD) models^39,40^, where the array of cells is only a discrete *version* of the spatially continuous RD model, diffusion parameters are typically constant - producing parametrically homogeneous arrays where only the voltage differences $\Delta V^{\left(i\right)}$ distinguishes the inner from the boundary cells. It has been shown that while the traveling front solution is robust in homogenous continuous systems, independent of the magnitude of diffusion coefficient D, the coupling strength in the discrete models (GJ conductivity in our model) is critical and could lead to different scenarios of propagation failures even in the simplest forms of the coupling functions^39,41^.

Our GJ-coupled cell has a unique form as a 2-d differential-difference FitzHugh-Nagumo class of ODE models^42^ in two respects:

- voltage kinetics $f\left(V_{m},I_{\text{ext}}\right)$ is essentially one-dimensional, while the slow variable $n_{gj}$ comes from GJ deactivation, and therefore acts on the coupling, diffusional term **but not** on the *own voltage dynamics* in first ODE,
- $n_{gj}$ is therefore not a recovery variable in its nature, since it scales the junctional voltage $V_{j}$, and due to the N-shaped nonlinearity it produces nonlinear GJ current terms $n_{gj}\left(V_{\text{neighbor}}-V_{m}\right)$, which may produce very different effects.

Consequently, we formulate the following properties of Eq. (3):

- The kinetics $f\left(V_{m},I_{\text{ext}}\right)$ in Eq. (3) can have only fixed-point solutions, which does not rule out periodic solutions in the coupled system, as a composite effect of two N-shaped nonlinearities $f+n_{gj}\Delta V$.
- The dynamical system in Eq. (3) is not excitable, meaning that solitary spikes are also not permissive, because the slow variable $n_{gj}$ is not a recovery variable of f, and the necessary dynamical structure is not present. This leaves bistability as the only instability originating in f, with propagating front as a possible non-trivial array solution.
- With $n_{gj}$ positive, the sign of the coupling term is defined by the sign of $\Delta V_{j}^{\left(i\right)}$. For inner cell in 1-d array, with initial ordering $V_{r}\ll V_{\text{own}}<V_{\text{dr}}$ the left voltage difference $\Delta V_{j}^{l}=V_{\text{dr}}-V_{m}$ drives outward, depolarizing current and competes with $\Delta V_{j}^{r}$ driving smaller but negative current at the right GJ.
- For the range of physiologically observed glial depolarizations, not exceeding 15-20mV (even in seizures and spreading depressions), for simplicity we will ignore in the initial analysis $\tau_{gj}\left(V_{j}\right)$ voltage dependence. Instead, we assume a constant $\tau_{gj}$, corresponding to $0\leq V_{j}\leq 20\;mV$, which is the range of maximal GJ conductance, see Fig. 2D and 2E^28^.

### 3.2 Self-coupled cell (single active site)

We will explore perturbations of $V_{r}$ in *one inner cell* with the preceding, left $\left(i-1\right)$ neighboring cell depolarized and kept at $V_{\text{dr}}$, while the next, (i+1) cell to the right and the remaining ones up to the last one are initially at steady-state $V_{r}$, but dynamically evolving. For simplicity, we will keep the same resting state $V_{r}$ in all cells, as indicated before. How strong will be the propagation effect depends on (a) the separations of the fixed points, usually analyzed using $\left(V_{s}-V_{r}\right)$ distance^43,44^, and on (b) the slow deactivation governed by $n_{gj}$.

Very long $\tau_{gj}$, from hundreds of milliseconds to seconds warrants analyzing a single inner cell (*single active site*^45^) ignoring the much slower temporal evolution of the “tail” of 1-d array. An approach already employed in propagating front and solitary waves propagation in bistable media^43,45^. The junctions to the right with very small or zero $\Delta V_{j}$ at the onset of perturbation produces very small junctional current despite the highly conductive state of the GJ, Fig. 2E. In quantitative terms, the above means that $g\left(n_{gj}\right)$ at the left connection of the active site produces strongly nonlinear $I_{gjc}\left(V_{j}\right)$, bending toward $g_{\text{min}}V_{j}$ for larger $V_{j}$. On the right connection, junctions between the cells other than the active cell sense very small perturbation in the linear regime with $I_{gjc}\sim g_{\text{max}}V_{j}$, where $V_{j}$ is very small^45^, Fig. 2F.

To conclude on the model class, reducing the FitzHugh-Nagumo model Eq. (4), to a single cell, *self-coupled to fixed boundaries*, we do have a Bonhoeffer-Van der Pol (BvP) system:

(4) $$CdV/dt=f\left(V,I_{\text{ext}}\right)+g\left(n_{gj}\right)\Delta V_{j},\;(nA),\;dn_{gj}/dt=\left(n_{\infty }(V)-n_{gj}\right)/\tau_{gj},$$

where f has the cubic, N-shaped nonlinearity fulfilling the sufficient condition of non-monotonicity for bistability of f^46^. The additional condition of bounded perturbations^46^, is *a priori* granted in biological settings by reducing the perturbations $g\Delta V_{j}$ and $I_{\text{ext}}$ to experimentally observable.

Since the main analytical impact on the shape of f comes from the nonlinearity in $I_{\text{Kir}}$ given in (Janjic et. al, 2023)^11^, we copy the full expression here for convenience:

(5) $$I_{\text{Kir}}=zFP_{\text{Kir}}\left({\left[K^{+}\right]}_{i}-{\left[K^{+}\right]}_{o}e^{-\frac{dz\;V_{m}}{v_{S}}}\right)/\left(e^{\frac{-\delta \left(\Delta z_{B}+dz\right)\left(V_{m}-V_{12-Kir}\right)}{v_{s}}}+1\right)\;(nA),$$

where $\Delta z_{B}$ – charge equivalent of physiological block of Kir channels controls the qualitative shape of $I_{\text{Kir}}(V)$, Fig 6A, and the nature of rectification, with $P_{\text{Kir}}$ and dz producing additional shaping^11^. Expectedly, $I_{K2P}$ and $I_{\text{leak}}$ in Eq. (2) natively modulate the shape of the summary I-V curve, but present in much smaller proportions their effect on the N-shaped curve is much weaker, compared to Kir current which is dominant in amplitude.

The ranges of some of estimated passive parameters like: maximal conductances, input resistance, or the total membrane capacitance need numerical adjustments, when estimated without recordings from intact tissue. Such recordings from glia *always* “pick” a signal from an ***effective cell*** or an irregular assembly formed by the patched (measured) astrocyte and most likely parts of several GJ-connected immediate neighbors, due to very leaky membrane^3^ and very conductive GJs in nominal physiological conditions^3,14,15^, **see** Supplementary material S3, for illustration.

### 3.3 Main coupling approximation

Assuming the main kinetical transition in GJs, the voltage-dependent deactivation $n_{gj}$ is very slow compared to membrane time constants, $\tau_{m}\ll \tau_{gj}$, we modify Eq. (3) to obtain single-valued $g\left(n_{gj}\right)$*,,* i.e. a proper ODE usable in numerical phase-plane and bifurcation analysis:

(6) $$C_{m}dV_{m}/dt=f\left(V_{m},I_{\text{ext}}\right)+l_{\text{scal}}\;{\overset{‾}{g}}_{gj}n_{gj}\left(V_{\text{dr}}-V_{m}\right)+r_{\text{scal}}\;{\overset{‾}{g}}_{gj}n_{\text{max}}\left(V_{r}-V_{m}\right)\;(nA),\;dn_{gj}/dt=\left(n_{\infty }\left(V_{j}\right)-n_{gj}\right)/\tau_{gj}.$$

As discussed above, the left connection senses larger positive $\Delta V_{j}^{l}=V_{\text{dr}}-V_{m}$ operating within transition region of $g\left(V_{j}\right)$, and requires evaluating $g\left(n_{gj}\right)$ when solving Eq. (3). On the other hand, the junctions to the right are under very small $\Delta V_{j}^{r}=V_{m}^{r}-V_{m}$, with $V_{m}^{r}\approx V_{r}$ meaning the junction operates at the plateau of the activation curve where $n_{gj}$ stays at $n_{\text{max}}$ of the fully conducting state, over a broad $V_{j}$ range of up to 40 mV. The initial condition we need for this simplification requires that at onset of jump transition in Cell-1, Cell-2 is not strongly depolarized, satisfying $\left|V_{r}-V_{s}\right|/\left|V_{\text{dr}}-V_{r}\right|\gg 0$.

Assigning asymmetrical, unequal fractional parameters $l_{\text{scal}}$ and $r_{\text{scal}}$ should be considered a requirement in coupled glia, rather than an exception. With each astrocyte on average connecting approximately ten neighbors, and with diffusive, rather than directionally spreading of massive depolarizations, a cell near front propagating boundary is engaged by multiple neighbors. Such *volume effect* statistically means in any moment of time, more GJs are engaged than still “quiet”. This implicates that $l_{\text{scal}}>r_{\text{scal}}$ is a safer assumption. Within the few illustrative cases of numerical bifurcation analysis of Cell-2 presented later, we kept the fractions near $l_{\text{scal}}=0.8$, and $r_{\text{scal}}=0.2$.

## 4. Numerical bifurcation analysis and simulations

As indicated by feature analysis of GJ connections, on top of nonlinear effects introduced f, increased bifurcation complexity should be expected due to coupling. In the extended system in Eq. (6), which has been kept in $R^{2}$, the existing saddle-node bifurcation of $V_{r}$ in isolated, produced by increasing $I_{\text{ext}}$ cell is central, because physiologically, most neural cells need external current $I_{\text{ext}}$ as a principal perturbation to demonstrate transient responses. In that regard the cases we illustrate here are extensions, or a sort of *add-on* complexity brought by GJ coupling, arising from the composite effects of two N-shaped nonlinearities in the coupling term $n_{gj}\circ \Delta V$, hidden behind $f\left(V_{m},I_{\text{ext}}\right)$ in $V_{m}$ Eq. (6).

**NOTE** - The values of those parameters of $f\left(V_{m},I_{\text{ext}}\right)$ vector field of isolated cell which are manipulated within numerical analysis are given in the corresponding sections. Parameter ranges of the other parameters as well as the equations describing the glial currents have been reused as published with the isolated cell model^11^.

### 4.1 Case 1 - Saddle-node bifurcation of $I_{\text{ext}}$ with a fold of limit cycles window

A fold bifurcation of $V_{r}$ over variable $I_{\text{ext}}$ is presented for an inner coupled cell, coupled to fixed boundaries, resting states $V_{\text{dr}}^{l}=-20\;mV,\;V_{r}^{r}=-55\;mV$, to the left and right respectively, as outlined before.

**Figure 3. –:**
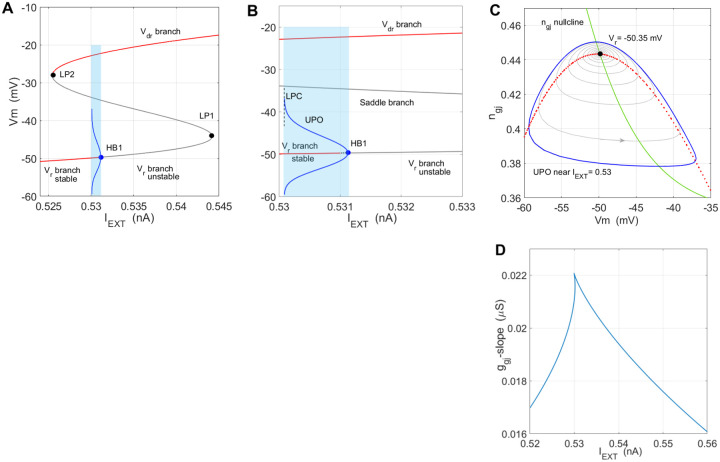
Saddle-node structure with an UPO window – **(A)** The generic fold-structure over $I_{\text{ext}}$ with a Hopf point HB1, and an unstable periodic orbit (UPO) window, shaded blue. (B) The UPO window (0.53, 0.531) enlarged, with the symmetrical blue curve outlining the amplitude of the UPO. This bifurcation structure limits notably the stable range of $V_{r}$ (red, stable branch) making it more sensitive to external depolarization. **(C)** A phase space view of the UPO (blue) near $I_{\text{ext}}=0.53$, around a stable focus at $V_{r}=-53.35\;mV$, with a period of approximately $T_{\text{UPO}}=303.8\;ms$. **(D)** Cusp curves are typical in search for biologically relevant two-parameter dependencies, where the other parameters like ${\overset{‾}{g}}_{gj}$, also produce fold-structures around $V_{r}$. Other changeable parameter values for (A-C): ${\overset{‾}{g}}_{gj}=0.0186\;\mu S,\;V_{\text{dr}}^{l}=-20\;mV,\;V_{r}^{r}=-55\;mV,\;P_{\text{Kir}}^{0}=8.2799\times 10^{-7}\;{\text{cm}}^{3}/\text{s},\;V_{12-\text{Kir}}=-55\;\text{mV},\;z_{BA}=-3.72,\;v_{s}=6,\;{\left[\;{\text{K}}^{+}\right]}_{o}=3.5\;mM,\;n_{gj-\text{min}}=0.35,\;l_{\text{scal}}=0.8,\;r_{\text{ccal}}=0.2$.

The generic saddle-node bifurcation structure of $I_{\text{ext}}$ observed in the isolated cell^11^ was altered by a very narrow window of unstable periodic orbit (UPO) cycles emerging from the Hopf point HB1 on the lower, stable $V_{r}$- branch, Fig. 3A, for $I_{\text{ext}}=0.531126\;nA$, and disappearing at the *limit point oof cycles* (LPC), Fig. 3B. Phase-plane example of the UPO is shown in Fig. 3C, with a period approximated to $T_{upo}=303.8\;ms$, for parameters given in Fig.3 caption.

The critical biophysical effect introduced by the coupling in this SN scenario is the notable shrinkage of the stable branch of $V_{r}$, ending at HB1, making the physiological RMV more prone to instability driven by $I_{\text{ext}}$.

In terms of phase-space geometry, these periodic orbits represent *small-amplitude* periodic orbits confined within the $V_{m}$ range of the lower branch, Fig. 3A and 3B, introducing very thin. 1pA wide repulsion window - a subset of $\left(V_{m},n_{gj}\right)\times [0.5301,0.5311]$, see (Izhikevich, 2007, Chapter 6.1.4)^47^ for surface geometry in $R^{2}\times C^{1}$. The UPO amplitudes saturate at the boundary of the narrow layer representing a *limit point of cycles*, LPC point, Fig. 9B.

**Figure 4. –:**
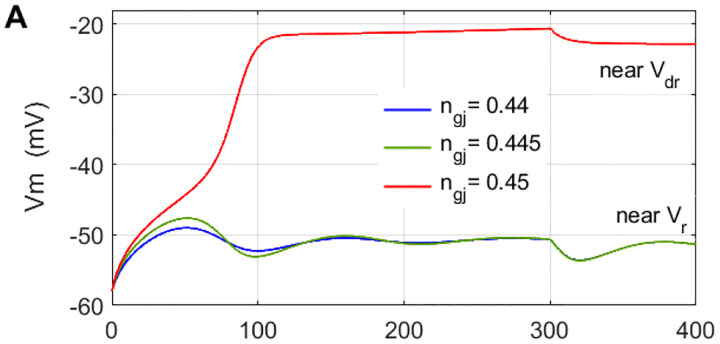

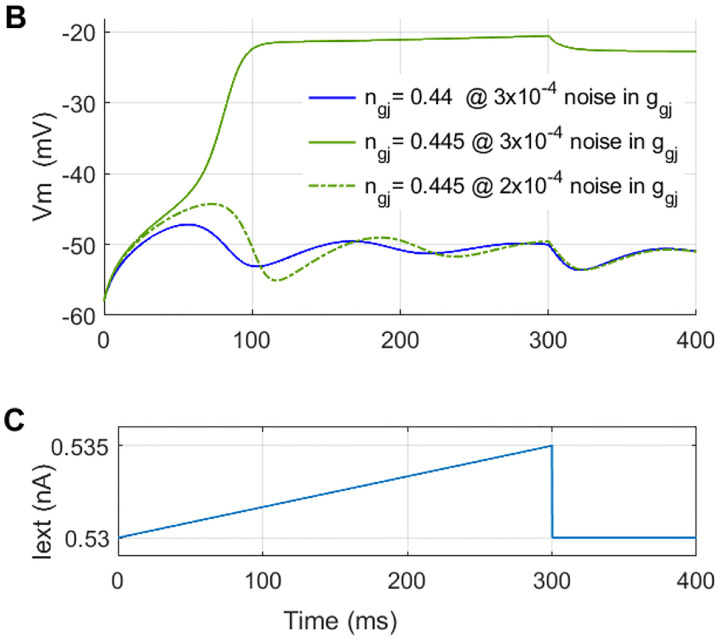
Noise-driven bistable switching near Hopf point – **(A)** Examples of three trajectories starting near $\left(-58\;mV,0.44\right)$. The initial voltage was kept $V_{m}^{0}=-58\;mV$, while $n_{gj}^{0}$ was moving the initial state away from the UPO (see Fig. 9C). The red curve shows the first switching from $V_{r}\to V_{\text{dr}}$, for the lowest $n_{gj}^{0}=0.45$, when depolarized by the ramp in (C). **(B)** same as in (A), with noise added to ${\overset{‾}{g}}_{gj}$ using normally distributed random numbers. Already for $n_{gj}^{0}=0.445$ (green graph) the noisy conductance takes the state to $V_{\text{dr}}$ which was not the case in (A). To show the additional sensitivity comes from the noise, the green dash-dotted line shows the solution for the same initial condition, but with decreased noise amplitude, not triggering switching to $V_{\text{dr}}$ **(C)** The ramp profile of $I_{\text{ext}}$ current used to depolarize the cell in simulations (A) and (B) moving the state through HB1. Precise comparison of trajectories with Fig. 9C above is not possible due to the ramp change of $I_{\text{ext}}$ in the latter case, changing the steady states and the nullclines.

The fold structure is generic when varying $n_{gj}$ as well, which is shown by the cusp diagram in $\left(I_{\text{ext}},{\overset{‾}{g}}_{gj}\right)$ plane, Fig. 4D. The case we are illustrating here lies near the ${\overset{‾}{g}}_{gj}$-branch of the cusp. A typical behavior of $V_{m}$ in the neighborhood of the UPO are pseudo-periodic transients of different lengths, depending on the value of the bifurcation parameter and the period $T_{\text{upo}}$. A variant of *slow ramp change of* $I_{\text{ext}}$ *through HB1* in either direction may provide long-enough dwelling* of the state near HB1, for $V_{m}$ to get affected by the repulsion of the UPO. Both packages, *XPPAUT* and *Matcont*^48^ detected HB1, while only the first one extended the fold branches up to the limit point of cycles, LPC.

Figure 4 illustrates the effect of noise in facilitating the saddle-node bistability switching near the UPO. Under a ramp profile of $I_{\text{ext}}$ going through HB1 from Fig. 9A and 9B, we show several transients where with other parameters unchanged, we varied $n_{gj}$ initial condition near the UPO

Introducing up to 3 × 10^−4^ (or 1.5%) random variation of the maximal GJ conductance ${\overset{‾}{g}}_{gj}=0.0186\;\mu S$, Fig. 4B, switched the state towards $V_{\text{dr}}$ already for initial conditions (-58mV,0.445) that were previously still within the basin of attraction of $V_{r}$, green solid-line traces in Fig. 4A. To verify the facilitated switching is an effect of parameter noise, we decreased the noise amplitude to 2 × 10^−4^ and expectedly the transient remained within the basin of $V_{r}$, with a slightly more pronounced variation of $V_{m}$, Fig. 4B, dash-dotted green curve.

**Figure 5. -:**
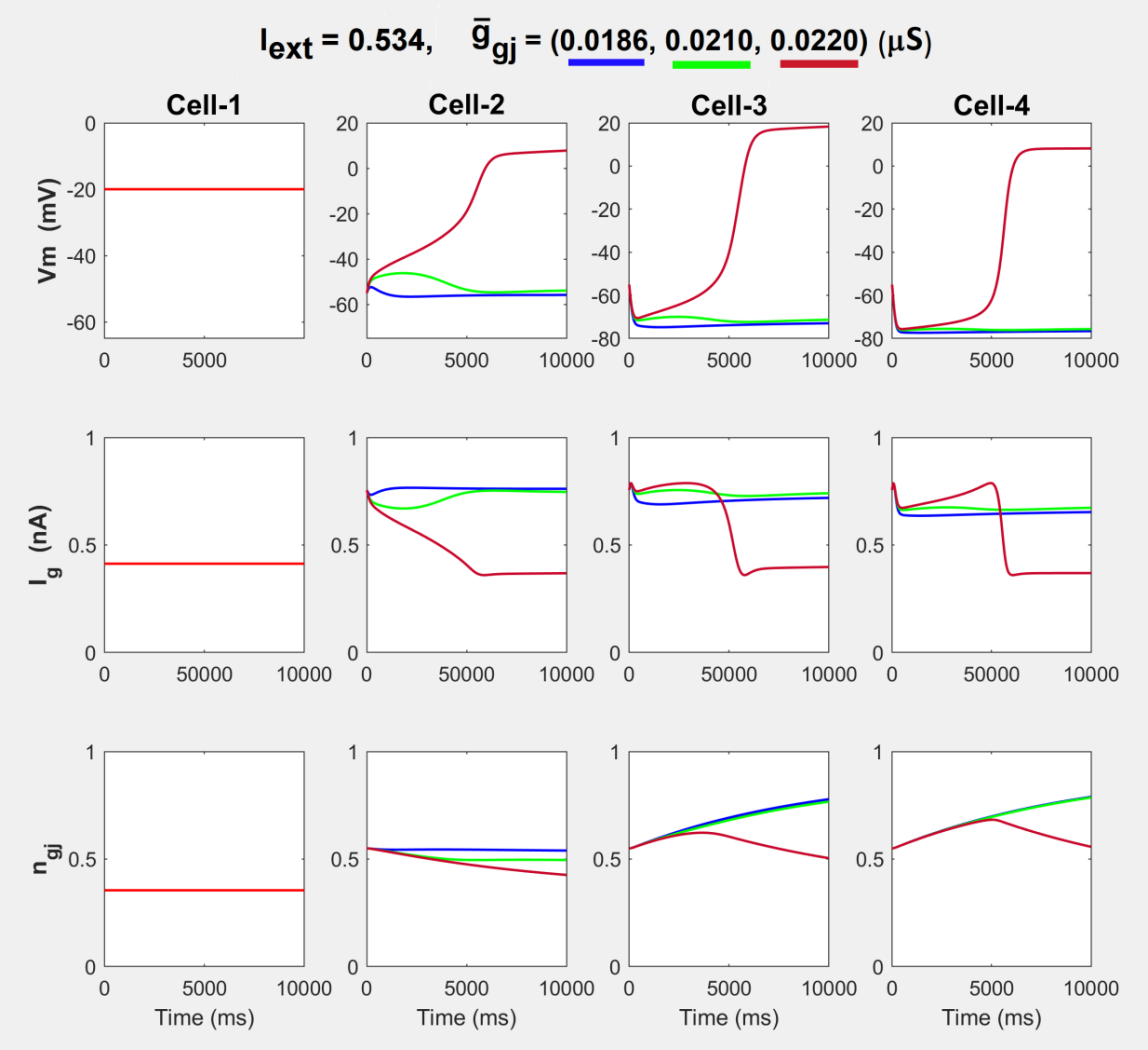
Temporal evolution of 1-d array of N=4 coupled cells with the Case-1 bifurcation structure in the inner cells, for $I_{\text{ext}}=0.534\;nA$ and $0.0186\;\mu S\leq {\overset{‾}{g}}_{gj}\leq 0.022\;\mu S$ showing propagation of the jump transition to $V_{\text{dr}}$ in the array, when Cell-1 is kept on $V_{\text{dr}}$ as a fixed boundary condition serving as array perturbation.

The results from the numerical simulations of the 1-d array of coupled cells, N=4, are presented in Figure 5. For constant $I_{\text{ext}}=0.534\;nA$, the coupling strength was gradually increased $0.0186\;\mu S\leq {\overset{‾}{g}}_{gj}\leq 0.022\;\mu S$, and color-coded temporal evolutions of $V_{m}$, total glial current $I_{\text{glia}}$ and gap-junctional voltage inactivation $n_{gj}$ are shown for 10 seconds. The array is perturbed by keeping the Cell-1 permanently at the UP-STATE, $V_{\text{dr}}=-20\;mV$, in bistability configuration. With $\tau_{gj}=100\;ms$, much slower evolution of $n_{gj}$ is a common feature of this model, evident in all $\tau_{gj}$ traces.

### 4.2 Case-2 – Enriched multistability with limit cycle bifurcations

Following bifurcation scenarios are relevant because the maximal GJ conductance ${\overset{‾}{g}}_{gj}$ is the sole bifurcation parameter, and they give birth to a stable periodic spiking in a measurable range of parameter change.

**Figure 6. –:**
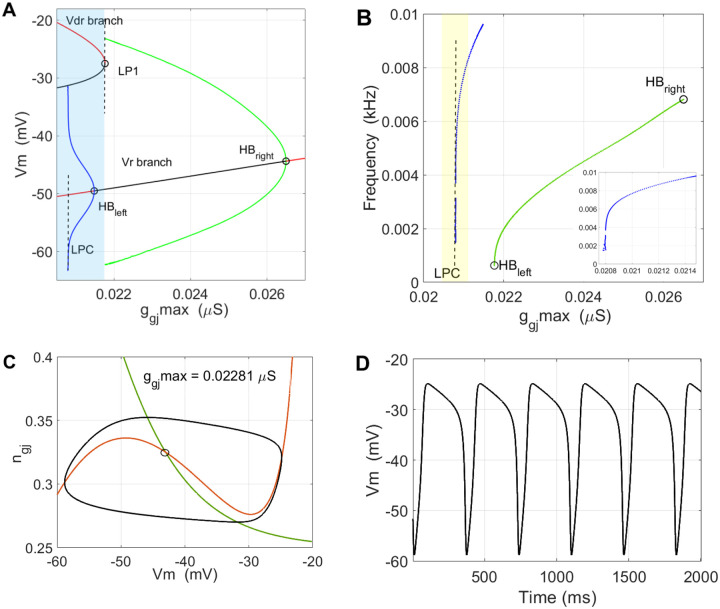
Large-orbit SPO meeting a “crisis” band of bistable behavior – **(A)** Bifurcation diagram $\left({\overset{‾}{g}}_{gj},V_{m}\right)$ showing 6nS wide window of ${\overset{‾}{g}}_{gj}$, where SPOs emerge through supercritical Hopf bifurcation along the $V_{r}$ branch by decreasing the slope conductance ${\overset{‾}{g}}_{gj}$ to the left of $HB_{\text{right}}$. Large-amplitude SPO disappears entering bistable switching window at LP1 of the fold structure of $V_{\text{dr}}$ branch (light blue shaded layer). **(B)** Frequency plot of both intervals of limit cycles, of the SPOs in green, and of the UPOs in blue. **(C)** and **(D)** Phase plane and timeseries example of a large-orbit SPO for ${\overset{‾}{g}}_{gj}=0.02281\;\mu S$. Other changeable parameter values for (A-C): $V_{\text{dr}}^{l}=-20\;mV,\;V_{r}^{r}=-55.4\;mV,\;P_{\text{Kir}}^{0}=8.2799\times 10^{-7}{\;cm}^{3}/s,\;V_{12-\text{Kir}}=-55\;mV,\;z_{BA}=-3.72,\;v_{s}=6,\;{\left[{\;K}^{+}\right]}_{o}=3.5\;mM,\;n_{gj-\text{min}}=0.25,\;n_{gj-\text{max}}=0.95,\;l_{\text{scal}}=0.8,\;r_{\text{ccal}}=0.2$.

Figure 6A describes the emergence of stable oscillations, over a measurable interval of ${\overset{‾}{g}}_{gj},5-6\;nS$ wide. They appear via super-critical Hopf bifurcation at $HB_{\text{right}},{\overset{‾}{g}}_{gj}=0.02649\;\mu S$ with the parabola extending toward lower values (green arcs) ending into a narrow *crisis window* Fig. 6A, shaded blue.

Within less than 1nS wide window along ${\overset{‾}{g}}_{gj}$ we have similar structure like in Case-1, dividing the $V_{m}$ range between **(1)** a band of UPOs at $V_{r}$ branch starting at $HB_{\text{left}},{\overset{‾}{g}}_{gj}=0.02148\;\mu S$ and ending on **LPC** at ${\overset{‾}{g}}_{gj}=0.02078\;\mu S$, and **(2)** a fold structure within $V_{\text{dr}}$ range. The arcs of Hopf parabola ends on LP1 point of the window.

The frequency plot on Fig. 6B shows frequency dependence of both periodic structures described in this scenario. The green curve, describing the SPOs, displays the known dependence for supercritical Hopf with frequency decreasing as ${\overset{‾}{g}}_{gj}$ approaches the LP1 limit point, reflecting the critical slowdown near a saddle onset. The blue curve, representing the fold of UPO limit cycles, Fig. 6B, yellow shaded band enlarged in the inset, shows a frequency scaling as $\text{frequency}\sim -\lambda_{1}/\text{log}\left(\tau *\left|{\overset{‾}{g}}_{gj}-{\overset{‾}{g}}_{gj}^{LPC}\right|\right)$ It decreases but remaining non-zero as ${\overset{‾}{g}}_{gj}\to {\overset{‾}{g}}_{gj}^{LPC}$, known to be a feature of saddle homoclinic bifurcation (SHO)^47^ which we did not detect numerically, see Fig. S4 in Supplementary Material 4, for the least-squares curve fitting.

Next, we present a small-amplitude *back-to-back SPO* bifurcation structure within the domain of the previous $\left({\overset{‾}{g}}_{gj},V_{m}\right)$ bifurcation diagram, where through a slightly different scenario, the nominal resting state $V_{r}$ switches to small-amplitude SPO by changing ${\overset{‾}{g}}_{gj}$, from either side. Increasing the ${\overset{‾}{g}}_{gj}$ conductance from the left, $V_{r}$ loses the stability undergoing a *fold limit cycle bifurcation*, while decreasing it from the right it undergoes an onset of stable spiking through a supercritical Hopf bifurcation.

**Figure 7. –:**
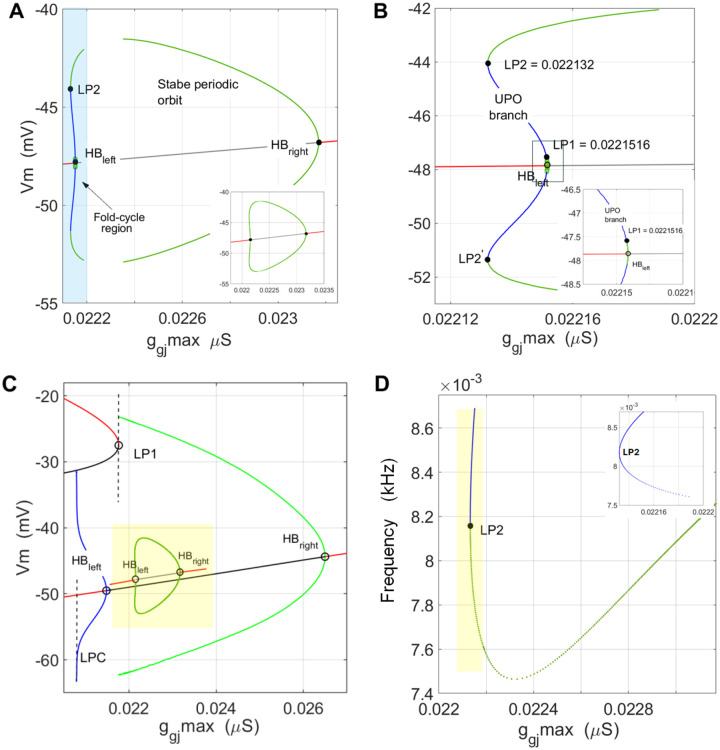
Back-to-back limit cycle bifurcation – **(A)** Bifurcation diagram $\left({\overset{‾}{g}}_{gj},V_{m}\right)$ showing a region of stable limit cycles (SPOs) about 1 nS wide, emerging through two different Hopf bifurcations, standard HB supercritical at $HB_{\text{right}}$ point and *fold cycle bifurcation* at $HB_{\text{left}}$ point. Running both continuations separately produced a closed loop, see inset. **(B)** The blue shaded region in (A), 0.1 nS wide expanded, illustrating that in fold cycle bifurcation structure the radius of the orbit undergoes a fold bifurcation^47^. **(C)** The small-orbit back-to-back SPO structure (shaded yellow) shown within the bifurcation picture of the previous, large-orbit SPO scenario. **(D)** Frequency plot in kHz, where the observed stable spiking has a non-zero frequency at onset which is in accordance with the properties of both transitions, supercritical Hopf and fold limit cycle^47^.

Figure 7 illustrates this back-to-back bifurcation structure. Both curves in Fig. 7A are obtained by periodic-orbit continuation from the left and right Hopf points, $HB_{\text{left}}$, or ${\overset{‾}{g}}_{gj}=0.02215175\;\mu S$ and $HB_{\text{right}}$ or ${\overset{‾}{g}}_{gj}=0.02317\;\mu S$. With suitably selected continuation step they typically connect each other, Fig. 7A inset. Increasing ${\overset{‾}{g}}_{gj}$ from the left (labeled as “$g_{gj-\text{max}}$” on the figures) the state passes through a very narrow “repelling layer” that is actually a fold, similar to the one exhibiting UPO orbits in Case-1, but even narrower in size, ~2 × 10^−5^, or 0.02nS, Fig. 7B. - effectively a boundary line of onset of the small-amplitude SPO with nonzero amplitude and frequency, Fig. 7D. The blue-stripe enlarged in Fig. 13B and the inset, between LP1 and LP2 pairs of points on each arc, show a fold structure due to which this limit cycle bifurcation structure at $HB_{\text{left}}$ is named *fold limit cycle* bifurcation, or *saddle-node of limit cycles*^47^. The non-zero terminating frequency of the UPOs, the blue curve, Fig. 7D and inset, additionally qualifies this fold of UPO cycles as fold cycle bifurcation rather than subcritical Hopf bifurcation.

A key observation in both above scenarios is that decreasing the coupling conductance, to the right of Hopf points, $HP_{\text{right}}$ we observe the birth of oscillations, which may sound counterintuitive from biophysical perspective. Getting back to the cusp curve in Fig. 3D of the Case-1 parametrically very close scenario, reminds us the fold is generic over wide range of ${\overset{‾}{g}}_{gj}$ suggesting that weakening the coupling, or gradually *disconnecting glia* may bring us in the range of spiking instabilities. Even though we analyze here an ODE representing a single cell connected to fixed voltages, it is a novel possibility encountered in dynamical studies of glial membrane.

**Figure 8. -:**
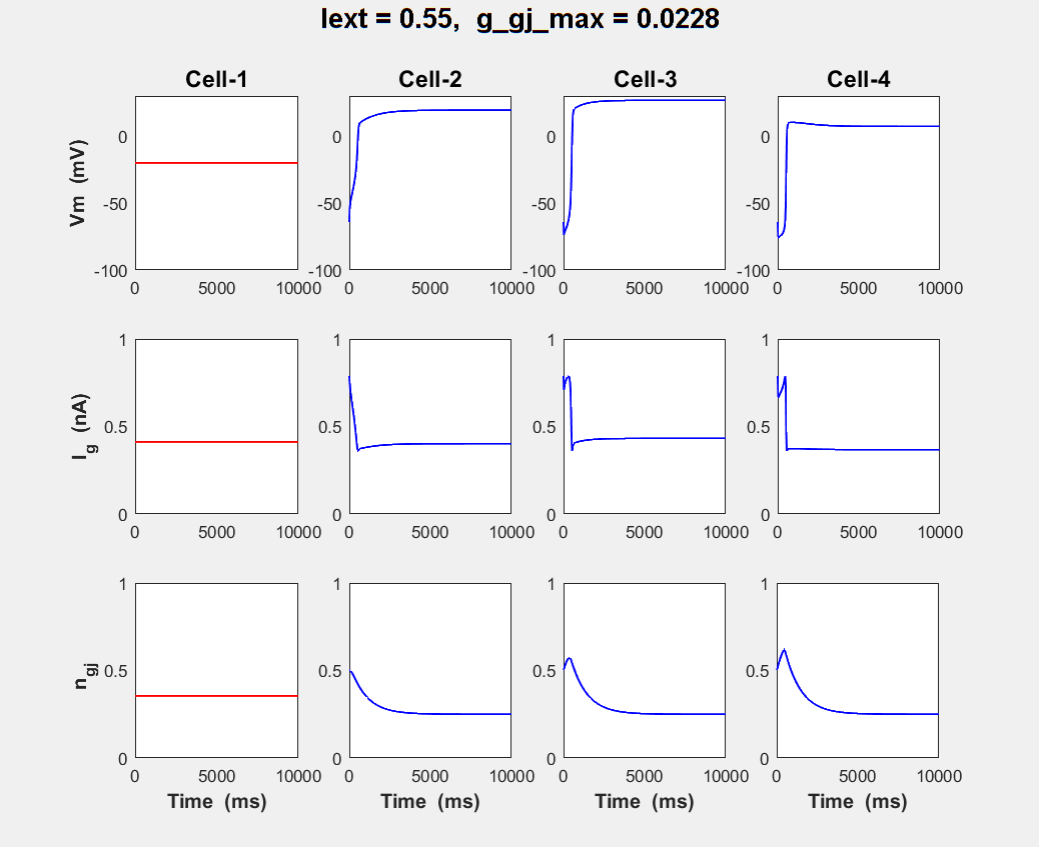
Temporal evolution of 1-d array of N=4 coupled cells, in the back-to-back SPO bifurcation scenario, for $I_{\text{ext}}=0.55\;nA$ and ${\overset{‾}{g}}_{gj}=0.0228\;\mu S$, within the range of the SPO, showing a very fast jump transition to $V_{\text{dr}}$ in the whole array, with Cell-1 kept on $V_{\text{dr}}$ as a fixed boundary condition serving as array perturbation. We stress here that ${\overset{‾}{g}}_{gj}=0.0228\;\mu S$ in array simulation does not matches exactly the parametric region in bifurcation diagram $\left({\overset{‾}{g}}_{gj},V_{m}\right)$ in Fig. 13A and 13B because here we simulate non-stationary ODEs due to coupling to a “live” Cell-3 and therefore shifting boundarv $V_{r}^{r}$.

We simulated the 4-cell array using parameters of the second of two SPO scenarios, with $I_{\text{ext}}=0.55\;nA$ and ${\overset{‾}{g}}_{gj}=0.0228\;\mu S$ in Figure 8. It corresponds to $\left({\overset{‾}{g}}_{gj},V_{m}\right)$ region of the large-orbit SPOs, close to the left end of the Hopf parabola. Typically observed behavior was a very fast jump $V_{r}\to V_{\text{dr}}$ suggesting that the effective coupling strength moves the state of inner, Cell-02 in the bistability region, corresponding to the blue shaded area in Fig. 6A. Differentiating feature of this scenario is more pronounced bistability in the array - for the same values of the fixed boundaries $V_{\text{dr}}^{l}=-20\;mV,\;V_{r}^{r}=-55\;mV$ like in the first SN scenario (Case −1), and very slightly perturbed ODE with ${\overset{‾}{g}}_{gj}>0.022\;\mu S$, exhibiting large-orbit SPO solution, the array displays much faster bistability propagation. Such higher propensity for front propagation is most likely result of the fold cycle effect.

Further parametric explorations and simulations that may result in criteria for observing of periodic solutions in 1-d array of coupled glia are complex challenges that we do not address here. The principal obstacle is the lack of coherent recordings of glial currents and GJ properties on the same set of cells. Numerical bifurcation analysis of such extended study will most likely engage two-parameter bifurcations lying probably near to the ones presented in Case-2.

## Discussion

So far, the highly conductive glial GJ connections have been modeled either as leaky Ohmic bridges, or open Nernst-Planck pores described by Goldman-Hodgkin-Katz equations^14^. In this study we tried to extend the present state of the art by introduction of voltage- and time-dependent GJ conductance. Implicated by strongly nonlinear $V_{j}$-dependence of junctional currents we introduced $V_{j}$-and time-dependent inactivation probability $n_{gj}\left(V_{j}\right)$ into a self-coupled single cell model. We argued that even though non-biological, the feedback-coupled glial cell through non-linear GJs to fixed voltages $V_{r}$ and $V_{\text{dr}}$ is a relevant model for numerical study of $V_{m}$ instabilities of $V_{r}$.

Both bifurcation structures analyzed suggest increased complexity and multistability when exploring the indicated ranges of $I_{\text{ext}}$ or slope conductance ${\overset{‾}{g}}_{gj}$, on top of the saddle-node transitions described in isolated cell model. In certain interval of ${\overset{‾}{g}}_{gj}$ range we observed for the first time periodic behavior exhibited by a glial model. All bifurcation pictures contained a band of UPOs on $V_{r}$ branch of the fold structure. In both Case-1 and Case-2 examples the UPO range represented a repelling structure either governing bistability switching, Fig. 3A and Fig. 6A, or the spiraling to small-amplitude periodic solutions in case of fold cycle, Fig. 7B. Further two-parameter bifurcation analysis near the fold limit points may uncover tangencies, homoclinic bifurcations and eventually second-order structures. To give legitimacy to such efforts experimental data are needed suggesting validated ${\overset{‾}{g}}_{gj}$ ranges, paired with external current $I_{\text{ext}}$ ranges.

Even though the demonstrated robust SPO behavior in our self-coupled cell needed decreasing ${\overset{‾}{g}}_{gj}$, rather than increasing, such scenarios may still have biological plausibility. There is strong experimental evidence from animal models that in chronic epilepsy, fully developed and prolonged seizures result in disconnection of glial cells in the hippocampus^49^, implicating also expressional changes of dominant Kir4.1 currents and real-time changes of gap-junctional properties^50^. We believe that the level of detail we incorporated in the self-coupled cell model could motivate modeling certain aspects of those experimentally observed alterations. In addition, failure of front propagation in arrays of Fitzhugh-Nagumo arrays has also been demonstrated in the limit of weak coupling^43^. In case of real cells, it probably requires an *attitudinal* shift that while erratic bistable behavior is commonly considered instability, a birth of stable spiking could be sort of multistability with certain biological purpose, even though it has not been observed in glial networks.

Our main motivation behind this experimentally “blind” modeling attempt was to further motivate suitably designed and targeted experimental studies that would measure transient responses of coupled glia, very likely to come from real-time voltage imaging on glia^51^, since it is seemingly impossible isolating them using whole-cell clamp protocols. Respecting the obstacles to reliably measure transient responses in leaky glial cells in intact tissue, we attempted filling some gaps in bringing the properties of time- and voltage-dependent GJ connections closer to future models of glial networks.

### Limitation of the coupled model –

Even the simplest array model, a linear 1-d array of cells, in principle is not solvable, apart from trivial cases not of interest in biologically motivated study. Analytical studies, reporting asymptotic analysis of simplified GJ connected 1-d^44^ arrays, illustrated that analyzing first inner cell of the array, should indicate the parameter ranges and nature of steady-state dependencies for observing jump transitions and front propagation.

Even though we kept the model of self-coupled cell 2-dimenional, both, the complex cell kinetics f and the coupling term taken together present many model parameters making the qualitative analysis of such ODE *over-defined* in parameters. For that reason, we stayed conservative with parameter ranges of all measurable parameters, avoiding speculative conclusions where aberrant parameter values in numerical continuations would have suggested potentially interesting bifurcation “twist”.

## Supplementary Material

Supplement 1

## Data Availability

The *XPPAUT*^52^ .ode files and parameter files of the bifurcation analysis, as well as 1-d array simulations in *Matlab* and simulations will be available in ModelDB at https://senselab.med.yale.edu/ModelDB, under model named “*Multistability in coupled astrocytes*”. The data dumps of particular bifurcation diagrams saved in AUTO / XPPAUT will be also available on request.

## Significance statement –

Transient changes in membrane voltage of glial cells may produce significant transient voltage difference between directly coupled cells. Nonlinear steady-state conductance of their interconnection elements, the gap junctions, introduce nonlinear current profiles which are very difficult to measure and quantitate using the available methods due to marked permeability of the junctions and leakiness of glial membrane in general. We propose a minimal model of glial membrane extended with a self-coupled feedback loop, which under realistic simplifying assumptions could serve for qualitative analysis of the impact of coupling, on the stability of resting membrane voltage.

## Footnotes

* We generalize here the scenario of $I_{\text{ext}}$ variation to include fluctuations as well, not necessarily requiring numerically ***strict*** monotonous change.
