# Supplementary material for: Membrane voltage multistability in coupled glial cells
Source: bioRxiv. 2026 May 8:2026.05.03.722503. Preprint. [Version 2] doi: 10.64898/2026.05.03.722503 (PMC13174323; doi:10.64898/2026.05.03.722503)
Supplement: Supplement 1 [file media-1.pdf]

SUPPLEMENTARY MATERIAL,

to accompany

Membrane voltage multistability in coupled glial cells

Predrag Janjic[1]✉, Dimitar Solev[1], Min Zhou[2], Ljupco Kocarev[1]

Supplementary Material S1 – Gap junctions and gap junction channels

Gap junctions (GJs) are formed by clusters of hundreds up to around two thousands of gap junction channels (GJCs), in a single membrane cluster[1] or a plaque, Fig. S1C. Each plaque represents one of multiple connections between two cells, or electrically one high-conductance junction. A single conductive GJ channel (a single GJC) between the two glial cells is formed when a pair of channel units (*connexons*), one at each side, are *docked* (axially aligned), Fig. 2B, to form a single conductive pore/path. Each connexon may have more than one conductive state, modulating complex gating transitions in some variants. The *Connexins* (abbreviated Cx), are the family of proteins building the channel pore on each side. Figure S1B shows the elements of this structural hierarchy. They form either *homotypic* GJCs, where both channels are made by the same connexin subtype, or *heterotypic* channels assembled of two different connexins, which way different GJCs display different conductive properties.

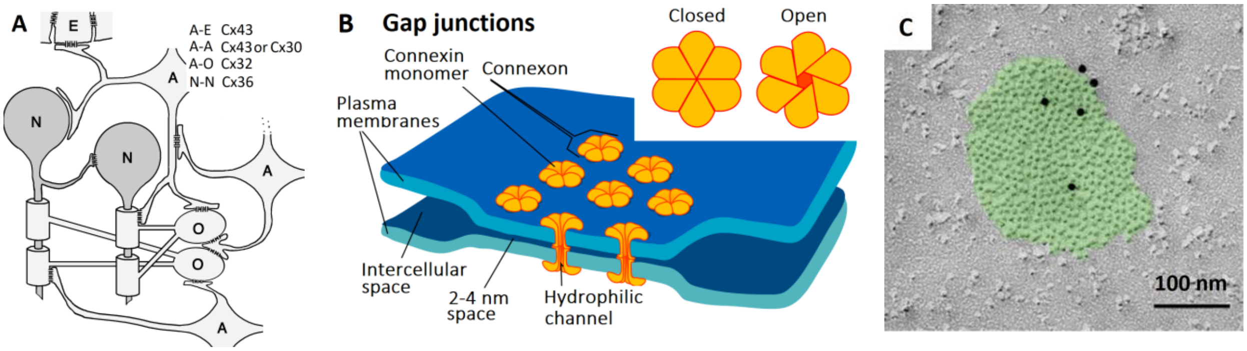

Figure S1. - Gap junctions (GJ) and gap junction channels (GJCs) – **(A)** Depending on the connection, astrocytes assemble different GJC combinations. Most typically, astrocytes are interconnected between themselves, by homotypic (uniform) Cx43-Cx43 or Cx30-Cx30 GJCs. Different cell designations mean: "A" astrocyte, "O" oligodendrocyte, "N" neuron, and "E" means endothelial cell of the blood vessels, or an ependymal cell at the brain external boundaries. Image from (Rash J. et al 2001)[2], with permission from *Society for Neuroscience*. **(B)** Hierarchical structure of the macroscopic gap junction, starting from Connexin protein units of a single channel, up to a physical cluster of docked channels forming one junction out of many. An open GJC pore is $\sim 2nm$ wide, which is 5-10 time wider than the pore in most of voltage-gated ion channels. The drawing adapted from Wikipedia, illustrated by Mariana Ruiz Villarreal. **(C)** Freeze-fraction electron micrograph of a plaque of channels forming a single gap junction connection, as they appear on the cell surface of one of the connecting astrocytes (while the other has been experimentally detached). Black dots are gold particles which appear as experimental artefact of the labeling method. Such structure may contain up to a few thousands channel pores. Image taken from J.C. Recktenwald, PhD thesis, 2020, University of Saarland, Homburg/Saar, Germany.

Sizes and channel densities within a single glial gap junction[3] suggest biophysical complexity that cannot be conceptualized with a single channel pore, Fig. S1C. For more structure-function details on GJ connections see (Sosinsky G., 2000)[4].

Supplementary Material S2 – **Equivalent circuit of recording setup from two GJ-connected cells**

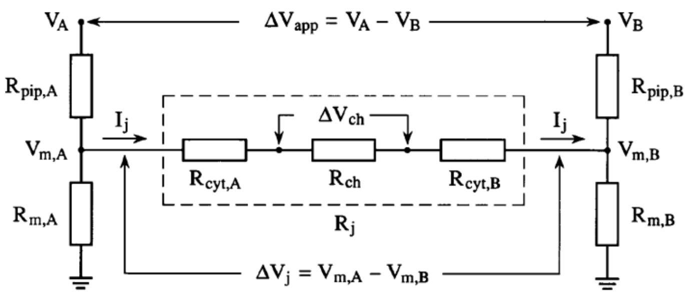

Figure S2. – Equivalent resistive circuit of double whole-cell recording setup – Each electrode, at V_A and V_B, introduces a serial pipette resistance R_{pip} and a branching point to the current path through the GJ. Apart from the cell input resistance R_m, the passive properties of the junction define *cytoplasmic access resistance* R_{cyt} in addition to the R_{ch}, the total Ohmic resistance of the cluster of GJ channels in *all junctions*. That sum is represented by a single resistor R_j since R_{cyt} and R_{ch} are inseparable. Designations adopted from the circuit in Ref. [5]

In terms of the equivalent resistive circuit diagram, generic for any two-electrode setup on two directly connected cells[5], Fig. S2, each of the electrodes A and B measures the voltage drop over the divider $R_{pip} + R_m$ typical for a whole-cell voltage clamp. In addition, the net current through gap junction(s) I_j, proportional to $V_{A,B}R_m \,/\, (R_{pip} + R_m\,)$ *sees* junction-specific cytoplasmic access resistance R_{cyt} in addition to R_{ch}, the total resistance of the plaque as a 2-terminal Ohmic element. The R_{cyt} resistance physically does not simply represent a fraction of the whole cell input resistance R_m, but specifically defined resistance by the passive resistive properties of the junctional plaque. R_{cyt} is not known since it is impossible to measure or sum up for all junctions, due to its size dependence[6]. Yet, it effectively introduces R_{cyt}/R_m fractional effect(s) on V_A, and V_B, or the *apparent voltage* difference $\Delta V_{app} = V_A - V_B$ that polarizes the junction(s). For more details on both, static and kinetic effects of the uncertainty in R_{cyt} and R_{ch}, see Wilders and Jongsma, 1992[5]. Being impossible to experimentally distinguish the contributions of R_{cyt} and R_{ch} we are passing to R_j of a whole junction, as a "symmetrical" resistance seen form either of the cells (to be further justified below). Apart from the artefactual resistances, we define the actual *transjunctional voltage* as $V_j = V_{m,A} - V_{m,B}$, by convention taken positive as $(V_{m,OTEHR} - V_{m,OWN})$. In the rest of our model development, we will also pass from apparent resistance to conductances, as conventional in conductance-based description of the neural circuits.

Supplementary Material S3 – **Adjusting maximal Kir permeability in *effective cell cluster***

Figure S3 illustrates the effect of a severalfold increase of Kir channel maximal NP permeability P_{Kir}^{0} on the shape of total glial current, in case of pronounced rectification, very negative Δz_B (see description of Kir current[7], or Eq. (5) main text. This way we try to get closer to the actually measured Kir conductances, $\bar{g}_{Kir}^{c}$ – the clamped cell with a fraction of active GJs to the immediate neighbors, defining an effectively measured cell cluster.

The I_{Kir} profile (Figure S3A, black) displays close to 2.2-fold increase in P_{Kir}^{0} compared to the value obtained in the model of an isolated cell[7]. In Fig. 8B a family of I_{Kir} profiles is illustrated for P_{Kir}^{0} in a rather wide range, between 1e-07 and 9e-07 cm^3/s, where the upper range should describe channel densities in the glial end-feet and the processes enwrapping the synapses[8]. Note that even the lowest value of P_{Kir}^{0} (bottom curve) produces inflection in I_{glia} curve which as a nonlinearity may give rise to oscillatory behavior.

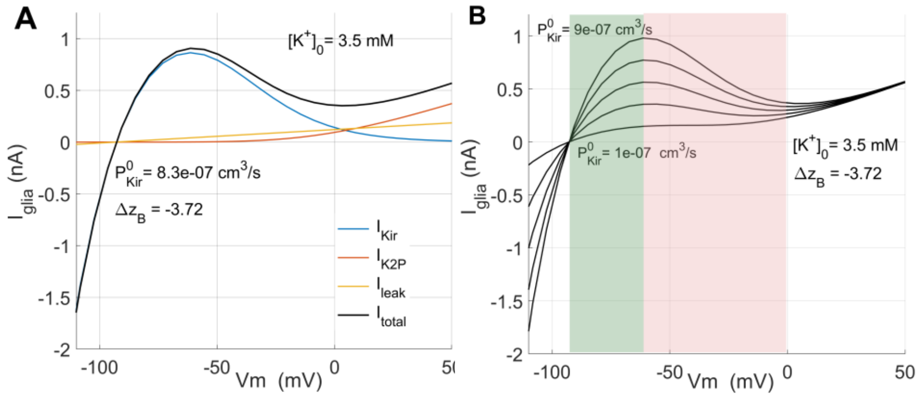

Figure S3. – **I-V profiles of $\boldsymbol{I_{glia}}$ for larger $\boldsymbol{P_{Kir}}$, (A)** Example of steady state I-V profile of glial current, with a 2.2-fold increase of P_{Kir}^{0} in Nernst-Plack description, Eq. (6), compared to (Janjic et al., 2023)[7], with $\Delta z_B = -3.72$. **(B)** Family of I-V curves, for P_{Kir}^{0} in the range 1e-07 to 9e-07 cm^3/s. The red-marked region is the range showing strongest nonlinear effect, where I_{kir} develops the N-shape.

Let us stress that the P_{Kir} range on Fig. S3B has been chosen to reflect the densities of channels suggested by voltage-imaging and expressional studies, rather than to construct curves with a sharper N-shape. As a sanity check, the equivalent slope conductance of the almost linear part of the curve, between -110 and -60 mV, would be close to **50 nS** which is well into the $\bar{g}_{Kir}^{c}$ ranges estimated from a single-cell *in situ* recordings with a cell capacitance in 25-50 pF range.

Apart from the change in the nonlinearity of the current profile, it is important to notice that the negative slopes, V_m range negative to V_r, appear steeper making the depolarization induced transitions more likely. In other words, smaller depolarizations will move the state from the positive I-V slope (the nominal, green-shaded WR profile) over the hump to the negative slope *transitory range* (red-shaded range), knocking the cell to the upper branch of the fold curve, and the depolarized state V_{dr}.

Supplementary Material S4 - Nonlinear Least Squares curve fitting of UPO frequency dependence on $\bar{g}_{gj}$ in Case-2 bifurcation scenario

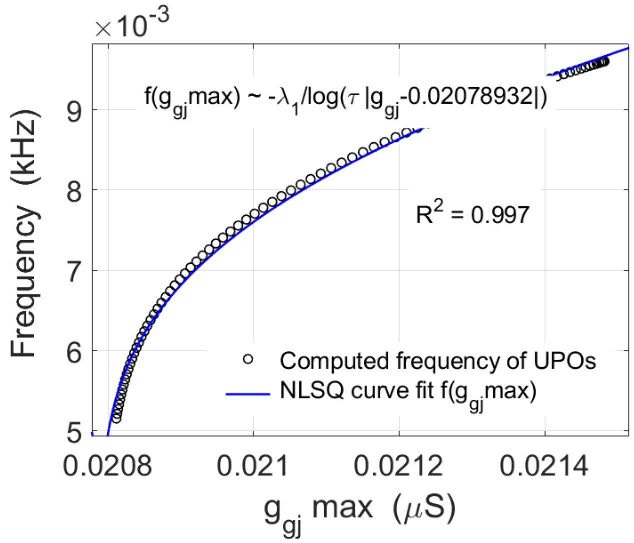

Figure S4 - UPO Frequency dependence on $\bar{g}_{gj}$ - The UPO curve approaches zero as $\lambda_1/\log(\tau * |\bar{g}_{gj} - \bar{g}_{gj}^{LPC}|)$ as $\bar{g}_{gj} \to \bar{g}_{gj}^{LPC}$, see inset, where b represents the bifurcation parameter $\bar{g}_{gj}$, known to be a feature of saddle homoclinic bifurcation (SHO)[9] scenario which we did not detect numerically. In such scenario $\lambda_1 = 0.04242$ should approximate the positive (unstable) eigenvalue of the saddle (at V_{dr} branch), which in this case a saddle number $(\lambda_1 + \lambda_2)$ less than zero corresponding to a band of UPOs. This suggest the possible origin of the corresponding bifurcation picture, Fig. 12A (main text), where the fold and UPO structures could be the "ruins" of SHO structure collapsing when meeting an SPO domain at LP1.

References

1 G. E. Sosinsky and B. J. Nicholson, "Structural organization of gap junction channels," Biochim Biophys Acta **1711** (2), 99–125 (2005).

2 John E Rash, Thomas Yasumura, F Edward Dudek, and James I Nagy, "Cell-specific expression of connexins and evidence of restricted gap junctional coupling between glial cells and between neurons," Journal of Neuroscience **21** (6), 1983–2000 (2001).

3 S. Aten, C. M. Kiyoshi, E. P. Arzola, J. A. Patterson, A. T. Taylor, Y. Du, A. M. Guiher, M. Philip, E. G. Camacho, D. Mediratta, K. Collins, K. Boni, S. A. Garcia, R. Kumar, A. N. Drake, A. Hegazi, L. Trank, E. Benson, G. Kidd, D. Terman, and M. Zhou, "Ultrastructural view of astrocyte arborization, astrocyte-astrocyte and astrocyte-synapse contacts, intracellular vesicle-like structures, and mitochondrial network," Prog Neurobiol **213**, 102264 (2022).

4 Gina Sosinsky, "Gap junction structure: New structures and new insights", in *Gap Junctions - Current topics in membranes* (Elsevier, 2000), Vol. 49, pp. 1–22.

5 R. Wilders and H. J. Jongsma, "Limitations of the dual voltage clamp method in assaying conductance and kinetics of gap junction channels," Biophys J **63** (4), 942–953 (1992).

6 H. Jongsma, R. Wilders, A.C.G. van Ginneken, and M.B. Rock, "Modulatory effect of the transcellular electrical field on gap junctional conductance", in *Biophysics of Gap Junction Channels*, edited by C. Peracchia (CRC Press, Boca Raton, FL, 1991), pp. 163–172.

7 P. Janjic, D. Solev, and L. Kocarev, "Non-trivial dynamics in a model of glial membrane voltage driven by open potassium pores," Biophys J **122** (8), 1470–1490 (2023).

8 M. Armbruster, S. Naskar, J. P. Garcia, M. Sommer, E. Kim, Y. Adam, P. G. Haydon, E. S. Boyden, A. E. Cohen, and C. G. Dulla, "Neuronal activity drives pathway-specific depolarization of peripheral astrocyte processes," Nat Neurosci **25** (5), 607–616 (2022).

9 E.M. Izhikevich, *Dynamical Systems in Neuroscience - The Geometry of Excitability and Bursting*. (The MIT Press, Cambridge, Massachusetts, 2007).
